# Statistical Emulation of Winter Ambient Fine Particulate Matter Concentrations From Emission Changes in China

**DOI:** 10.1029/2021GH000391

**Published:** 2021-05-01

**Authors:** Luke Conibear, Carly L. Reddington, Ben J. Silver, Ying Chen, Christoph Knote, Stephen R. Arnold, Dominick V. Spracklen

**Affiliations:** ^1^ Institute for Climate and Atmospheric Science School of Earth and Environment University of Leeds Leeds UK; ^2^ College of Engineering, Mathematics and Physical Sciences University of Exeter Exeter UK; ^3^ Faculty of Medicine University of Augsburg Augsburg Germany

**Keywords:** air quality, China, emissions, emulator, machine learning, particulate matter

## Abstract

Air pollution exposure remains a leading public health problem in China. The use of chemical transport models to quantify the impacts of various emission changes on air quality is limited by their large computational demands. Machine learning models can emulate chemical transport models to provide computationally efficient predictions of outputs based on statistical associations with inputs. We developed novel emulators relating emission changes in five key anthropogenic sectors (residential, industry, land transport, agriculture, and power generation) to winter ambient fine particulate matter (PM_2.5_) concentrations across China. The emulators were optimized based on Gaussian process regressors with Matern kernels. The emulators predicted 99.9% of the variance in PM_2.5_ concentrations for a given input configuration of emission changes. PM_2.5_ concentrations are primarily sensitive to residential (51%–94% of first‐order sensitivity index), industrial (7%–31%), and agricultural emissions (0%–24%). Sensitivities of PM_2.5_ concentrations to land transport and power generation emissions are all under 5%, except in South West China where land transport emissions contributed 13%. The largest reduction in winter PM_2.5_ exposure for changes in the five emission sectors is by 68%–81%, down to 15.3–25.9 μg m^−3^, remaining above the World Health Organization annual guideline of 10 μg m^−3^. The greatest reductions in PM_2.5_ exposure are driven by reducing residential and industrial emissions, emphasizing the importance of emission reductions in these key sectors. We show that the annual National Air Quality Target of 35 μg m^−3^ is unlikely to be achieved during winter without strong emission reductions from the residential and industrial sectors.

## Key Points

- We developed accurate and fast emulators to predict air quality in China from emission changes
- Winter ambient fine particulate matter concentrations were primarily sensitive to residential, industrial, and agricultural emissions
- The National Air Quality Target is unlikely to be met in winter without large emission reductions in the residential and industrial sectors

## 1 Introduction

Air pollution exposure is a leading public health problem in China (GBD 2017 Risk Factor Collaborators,  2018; Yin et al., 2020). Despite recent improvements in the air quality of China, air pollution exposure remains high, requiring further emission reductions to improve public health (Silver et al., 2020; Zhao et al., 2018). The impacts of potential emission changes on air quality can be determined through chemical transport model simulations. However, the large computational demands of these models limit the number of simulations and scenarios that can be feasibly investigated.

Machine learning models have been used for many different applications in air quality research (Bellinger et al., 2017; Karpatne et al., 2019; Reichstein et al., 2019; Rybarczyk & Zalakeviciute, 2018; Weichenthal et al., 2018). Specifically, machine learning models have been used as computationally efficient emulators of explicit chemical transport models, and used to explore uncertainties and sensitivities (Aleksankina et al., 2019; Carslaw et al., 2013; Lee et al., 2011, 2012, 2016; Ryan et al., 2018), to replace gas‐phase chemistry schemes (Keller & Evans, 2019), to predict aerosol mixing states (Hughes et al., 2018), and to predict air quality in India (Y. Chen et al., 2020) and the United Kingdom (Beddows et al., 2017). Y. Chen et al. (2020) trained machine learning models on simulation data from chemical transport models to predict changes in Indian air quality from emission changes, enabling extensive sensitivity analyses to be undertaken. Other machine learning approaches concerning Chinese air quality have been used to decouple the effects of meteorology and policies (Y. Zhang et al., 2020), to fuse model simulations with ground observations (Lyu et al., 2019), to optimize economic pathways to achieve air quality goals (Huang et al., 2020), and for the prediction of air pollution concentrations (G. Chen et al., 2018; Q. Li et al., 2018; Ma et al., 2019; Wei et al., 2020; Zhan et al., 2017).

Our aim was to develop computationally efficient emulators to predict winter ambient fine particulate matter (PM_2.5_) concentrations from emission changes in China. The emulators were trained on simulated data for January 2015 from chemical transport models, where emissions were varied in the following five key anthropogenic sectors: residential, industry, land transport, agriculture, and power generation. We used these emulators to explore how emission changes impacted winter PM_2.5_ exposure. To our knowledge, this is the first study using emulators to predict air quality in China from emission changes.

## 2 Methods

### 2.1 Simulator

Simulations were conducted using the Weather Research and Forecasting model online‐coupled with the Chemistry (WRFChem) version 3.7.1 (Grell et al., 2005; Skamarock et al., 2008). We refer to the chemical transport model (WRFChem) as the simulator. Simulations were for January 2015 with 1‐month spin‐up. The simulator domain covered China at 30 km (0.3°) horizontal resolution. The simulator setup is provided in Table S1 and described fully in our previous work (Reddington et al., 2019; Silver et al., 2020).

Anthropogenic emissions for China were provided by the Multi‐resolution Emission Inventory for China (MEIC) emission inventory for 2015 at 0.25 ° × 0.25 ° horizontal resolution (M. Li et al., 2017; MEIC Research Group & Tsinghua University, 2019; Zheng et al., 2018). Emissions were speciated for black carbon (BC), organic carbon (OC), PM_2.5_, coarse particulate matter (PM_10_), carbon monoxide (CO), ammonia (NH_3_), nitrogen oxides (NO_X_), sulfur dioxide (SO_2_), and non‐methane volatile organic compounds (VOC, Figures S1−S3). Anthropogenic emissions of methane inside China, and all anthropogenic emissions outside of China, were from the Emission Database for Global Atmospheric Research with the Task Force on Hemispheric Transport of Air Pollution (EDGAR−HTAP) version 2.2 for 2010 at 0.1° × 0.1° horizontal resolution (Janssens‐Maenhout et al., 2015). Sectoral emissions were provided for land transport, industry, residential energy use, power generation, shipping, aircraft, and agriculture. A diurnal cycle was applied to the anthropogenic emissions (Qi et al., 2017; Zheng et al., 2017).

Biomass burning emissions were from the Fire Inventory from National Center for Atmospheric Research (FINN) version 1.5 with a horizontal resolution of 1 km (Wiedinmyer et al., 2011). Biomass burning emissions were vertically distributed evenly throughout the model boundary layer. Emissions from vegetation were calculated online using the Model of Emissions of Gases and Aerosol from Nature (MEGAN, Guenther et al., 2006). Dust emissions were calculated online using the Global Ozone Chemistry Aerosol Radiation and Transport (GOCART) with the Air Force Weather Agency modifications (Legrand et al., 2019).

Gas phase chemistry was simulated using the extended Model for Ozone and Related Chemical Tracers (MOZART, Emmons et al., 2010; Hodzic & Jimenez, 2011; Knote et al., 2014). Aerosol physics and chemistry was simulated using the updated Model for Simulating Aerosol Interactions and Chemistry (MOSAIC) scheme with aqueous chemistry and the following four sectional discrete size bins: 0.039–0.156, 0.156–0.625, 0.625–2.5, and 2.5–10 μm (Hodzic & Knote, 2014; Zaveri et al., 2008). The secondary organic aerosol formation was based on an updated volatility basis set mechanism (Knote et al., 2015).

Microphysics were simulated using the Morrison two‐moment scheme (Morrison et al., 2009). Chemical initial‐ and boundary‐conditions were taken from operational simulations of the MOZART global chemistry transport model driven by the Goddard Earth Observing System Model (GEOS, National Center for Atmospheric Research, 2016). Meteorological initial‐ and boundary‐conditions were taken from the European Centre for Medium‐Range Weather Forecasts Re‐Analysis (ERA)‐Interim global product (Dee et al., 2011), on a N256 (∼35 km at the equator) grid at the surface and on a N128 (∼70 km at the equator) grid above the surface, and were updated every 6 h. WRF meteorology was nudged to these fields above the boundary layer.

### 2.2 Simulator Evaluation

Simulator evaluation against measurements was conducted in our previous work (Reddington et al., 2019; Silver et al., 2020). Measurement data was taken from over 1,600 sites across China, Hong Kong, and Taiwan as detailed in Silver et al. (2018). The normalized mean bias factor (NMBF) and the normalized mean absolute error factor (NMAEF) were used to evaluate the simulator (Yu et al., 2006). For January 2015, the simulator slightly overestimated PM_2.5_ concentrations across China (NMBF = 0.13 and NMAEF = 0.4), and the simulator slightly underestimated PM_2.5_ concentrations within the Guangdong‐Hong Kong‐Macau Greater Bay Area (GBA) in South Central China (NMBF = −0.04 and NMAEF = 0.11). Our evaluation shows that the simulator is able to predict the spatial pattern and magnitude of PM_2.5_ concentrations across China.

### 2.3 Emulator

Machine learning models can predict outputs based on statistical associations with inputs. We refer to the machine learning model developed here as the emulator. The aim of the emulators was to create computationally efficient proxies of the simulator that represent the relationships between changes in anthropogenic emissions and air quality. The emulators make predictions specific to their training data without explanatory knowledge (Deutsch, 2012; Pearl, 2019).

We developed emulators to predict how PM_2.5_ concentrations change as emissions from the residential (RES), industrial (IND), land transport (TRA), agricultural (AGR), and power generation (ENE) sectors change within mainland China. The emulator inputs were simulator data from 50 training runs and 5 test runs (Tables S2 and S3, respectively). The number of training runs (50) was determined as 10 times the number of inputs (five emission sectors) (Loeppky et al., 2009). Both the training and test simulator runs were designed from separate maxi‐min Latin hypercube space‐filling designs for each of the five inputs over 100,000 iterations (McKay et al., 1979). All anthropogenic species emitted for that sector were scaled by the corresponding factor between 0% and 150% for a given run. A different emulator was developed for each grid cell within China (15,278 grid cells in total) to capture the spatial distribution of the pollutants.

The emulator design was optimized using the Tree‐based Pipeline Optimization Tool (TPOT) (Le et al., 2020; Olson et al., 2016; Tran et al., 2016). The TPOT considered a range of emulator designs, evaluated each using 10‐fold cross validation, and optimized them for accuracy (Banzhaf et al., 1998; Fortin et al., 2012). Accuracy was measured as the coefficient of determination (*R* ^2^) and precision was measured as the root mean squared error (RMSE). The emulators focused on a Gaussian process regressor based on previous studies (Aleksankina et al., 2019; Beddows et al., 2017; Bellinger et al., 2017; Carslaw et al., 2013; Y. Chen et al., 2020; Lee et al., 2011, 2012, 2016; Rasmussen & Williams, 2006; Ryan et al., 2018; Rybarczyk & Zalakeviciute, 2018; Wild et al., 2020). The TPOT was used on 50 grid cells within China, selected using a reproducible random seed. The optimized emulator had a 10‐fold cross‐validation *R* ^2^ value of 0.9993. The optimized emulator design included input preprocessors (Yeo & Johnson, 2000), output preprocessors (zero‐mean and unit variance), and a Gaussian process regressor with a Matern 5/2 kernel (Conibear, 2020).

The emulators were used to predict output concentrations for configurations across the five emission sectors covering all permutations across a 0%–150% matrix of emission scaling factors at 10% increments. Exposures were estimated as population‐weighted concentrations using population count data for 2015 at 0.25° × 0.25° resolution obtained from the Gridded Population of the World, Version 4.11 (Center for International Earth Science Information Network & NASA Socioeconomic Data and Applications Center, 2018). National and regional analyses were conducted according to the following groupings (Figure S4): North China (Beijing, Tianjin, Hebei, Shanxi, and Inner Mongolia), North East China (Liaoning, Jilin, and Heilongjiang), East China (Shanghai, Jiangsu, Zhejiang, Anhui, Fujian, Jiangxi, and Shandong), South Central China (Henan, Hubei, Hunan, Guangdong, Guangxi, Hainan, Hong Kong, and Macau) including the GBA, South West China (Chongqing, Sichuan, Guizhou, Yunnan, and Tibet), and North West China (Shaanxi, Gansu, Qinghai, Ningxia, and Xinjiang), and the GBA individually.

### 2.4 Emulator Evaluation

**Figure 1:**
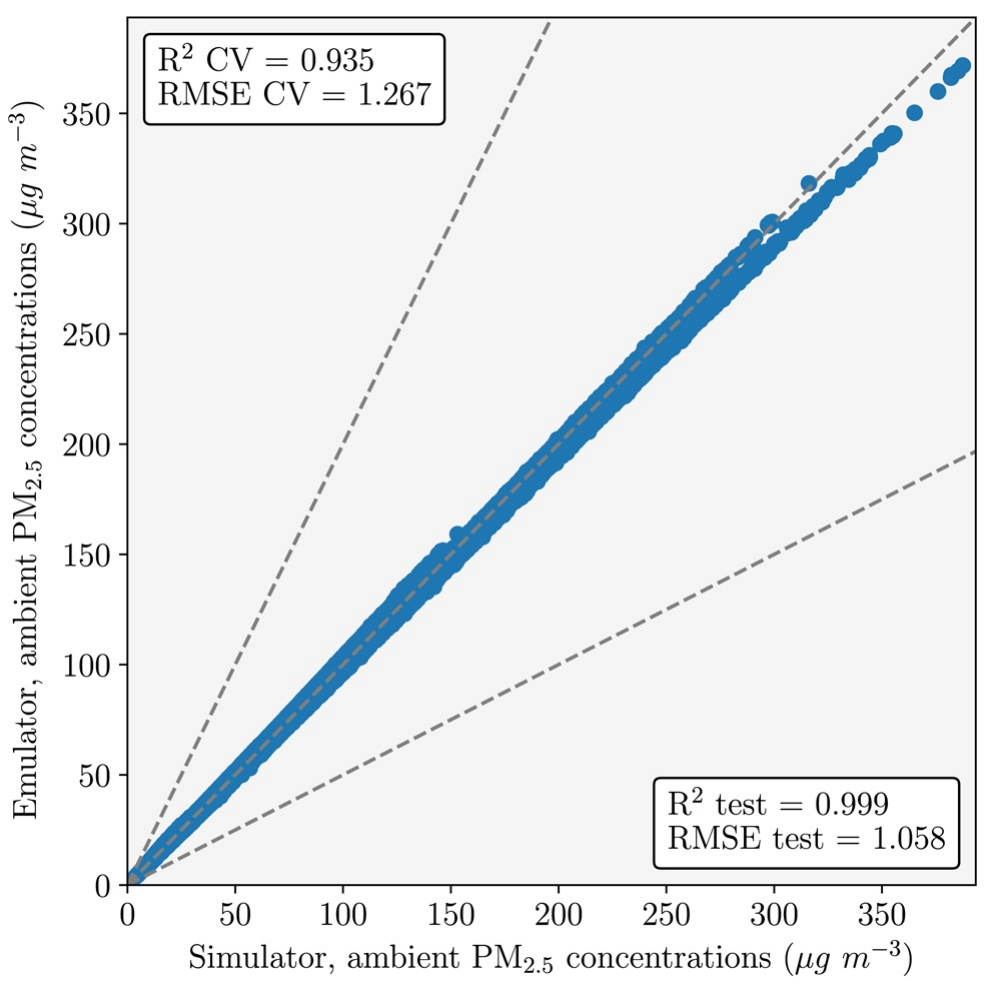
Emulator evaluation for ambient fine particulate matter (PM_2.5_) concentrations on the test (generalization) data. Evaluation metrics used were the coefficient of determination (*R* ^2^) and the root mean squared error (RMSE). Evaluation metrics given for both the test and training (cross‐validation, CV) data.

The emulators for all grid cells were evaluated on the training data using *k*‐fold cross validation (where *k* = 10) and separately on the held‐out test data (Figure 1). *K*‐fold cross validation randomly splits the training data into *k* smaller sets, trains the emulators on each of the *k−*1 smaller data sets, and then validates the emulators on the final held‐out data. The performance measures were then averaged across all the *k*‐fold cross validations.

Across all emulators, the mean 10‐fold cross validation *R* ^2^ values was 0.935 and the mean RMSE value was 1.267 μg m^−3^. For the test data, the *R* ^2^ value was 0.999 and the RMSE value was 1.058 μg m^−3^. This means that the emulator generalized well to new data and predicted 99.9% of the variance in PM_2.5_ concentrations for a given emission configuration.

### 2.5 Sensitivity Analysis

Global sensitivity analyses were performed on the emulators using a Saltelli sampler and a Sobol analyzer (Iooss & Lemaître, 2015; Kennedy & O’Hagan, 2000; Saltelli et al., 2010; Sobol, 2001). First‐order, second‐order, and total sensitivity indices were calculated for each input and output. First‐order sensitivity indices measure the contribution to the output variance by a single input individually. Absolute first‐order sensitivity indices are the first‐order sensitivity indices multiplied by the baseline PM_2.5_ concentrations. Second‐order sensitivity indices measure the contribution to the output variance caused by the interaction of two inputs. Total sensitivity indices measure the contribution to the output variance caused by an input, including both first‐order effects and all higher‐order interactions. The sensitivity indices for all emission sectors sum to 1 for each grid cell and were shown as percentages. The sensitivity indices were estimated based on 12,000 emulator runs per grid cell, decided upon through Equation 1, where *N* was 1,000 and *D* was the number of inputs (Kennedy & O’Hagan, 2000; Saltelli et al., 2010).

(1) Emulatorruns=N×(2D+2)

## 3 Results and Discussion

### 3.1 Baseline Ambient PM_2.5_ Concentrations

**Figure 2:**
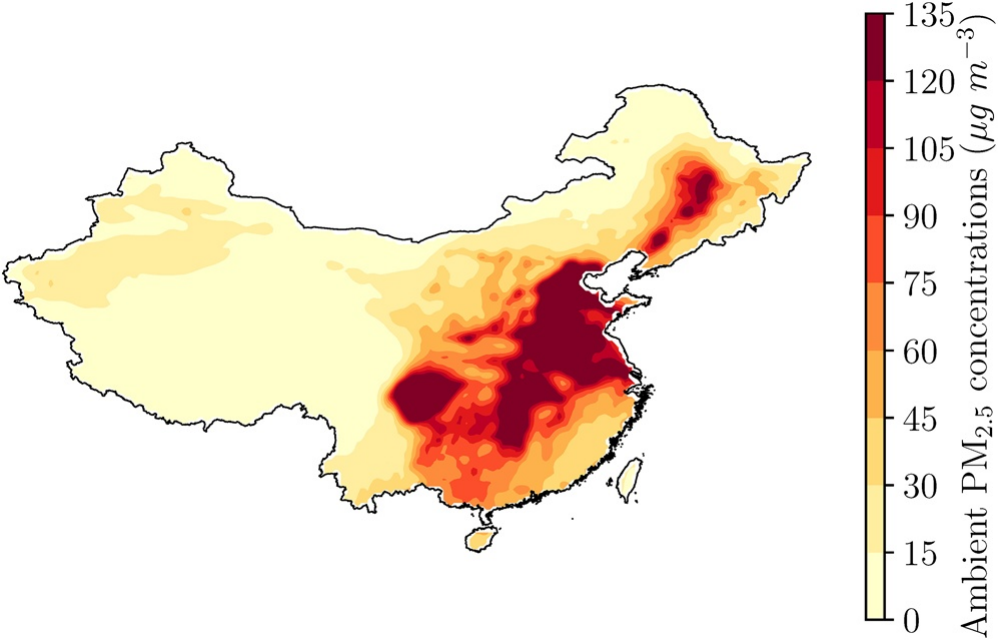
Emulator predictions of monthly‐mean (January 2015) ambient fine particulate matter (PM_2.5_) concentrations for the baseline scenario with all emission sectors at 100% across China.

The emulator results and discussion are monthly‐means for January 2015. PM_2.5_ concentrations from the emulator baseline (i.e., all emission sectors at 100%) are shown in Figure 2 and average PM_2.5_ exposures are given in Table 1. PM_2.5_ exposures are above 100 μg m^−3^ in China overall, driven by high exposure over North, South Central, South West, and East China, with lower exposure in the GBA, North West China, and North East China.

**Table 1:** Emulator Baseline Monthly‐Mean (January 2015) Ambient Fine Particulate Matter (PM_2.5_) Exposure

Baseline	China	GBA	North China	North East China	East China	South Central China	South West China	North West China
Ambient PM_2.5_ exposure (μg m^−3^)	104.4	54.8	122.2	80.8	104.1	111.5	116.6	50.2

### 3.2 Key Sensitivities of Ambient PM_2.5_ Concentrations to Emission Changes

Table 2 shows the first‐order sensitivities for PM_2.5_ concentrations for each of the five emission sector inputs. Across China, PM_2.5_ concentrations are most sensitive to residential emissions (64%), then approximately equally sensitive to industrial (16%) and agricultural emissions (14%). In all regions, PM_2.5_ concentrations are primarily sensitive to residential emissions (51%–94%). North West China has approximately equal sensitivity to industrial (23%) and agricultural emissions (22%). In South West China, agricultural emissions (24%) dominate over land transport (13%) and industrial emissions (7%). In the GBA, North China, North East China, East China, and South Central China industrial emissions (5%–31%) dominate over agricultural emissions (0%–11%). In all regions except in South West China, sensitivities of PM_2.5_ concentrations to land transport emissions are under 5%. Sensitivities of PM_2.5_ concentrations to power generation emissions are all under 3%. Second‐order sensitivities, which estimate sensitivities from input interactions, are all less than 1%.

**Table 2:** The First‐Order Sensitivities (%) for Ambient Fine Particulate Matter (PM_2.5_) Concentrations From the Emulator in January 2015 Per Region and Emission Sector of Residential (RES), Industry (IND), Land Transport (TRA), Agriculture (AGR), and Power Generation (ENE)

		China	GBA	North China	North East China	East China	South Central China	South West China	North West China
First order (%)	RES	64	56	73	94	77	81	52	51
	IND	16	31	20	5	18	13	7	23
	TRA	4	2	1	1	1	1	13	3
	AGR	14	11	4	0	3	4	24	22
	ENE	1	0	3	1	0	0	1	1

**Figure 3:**
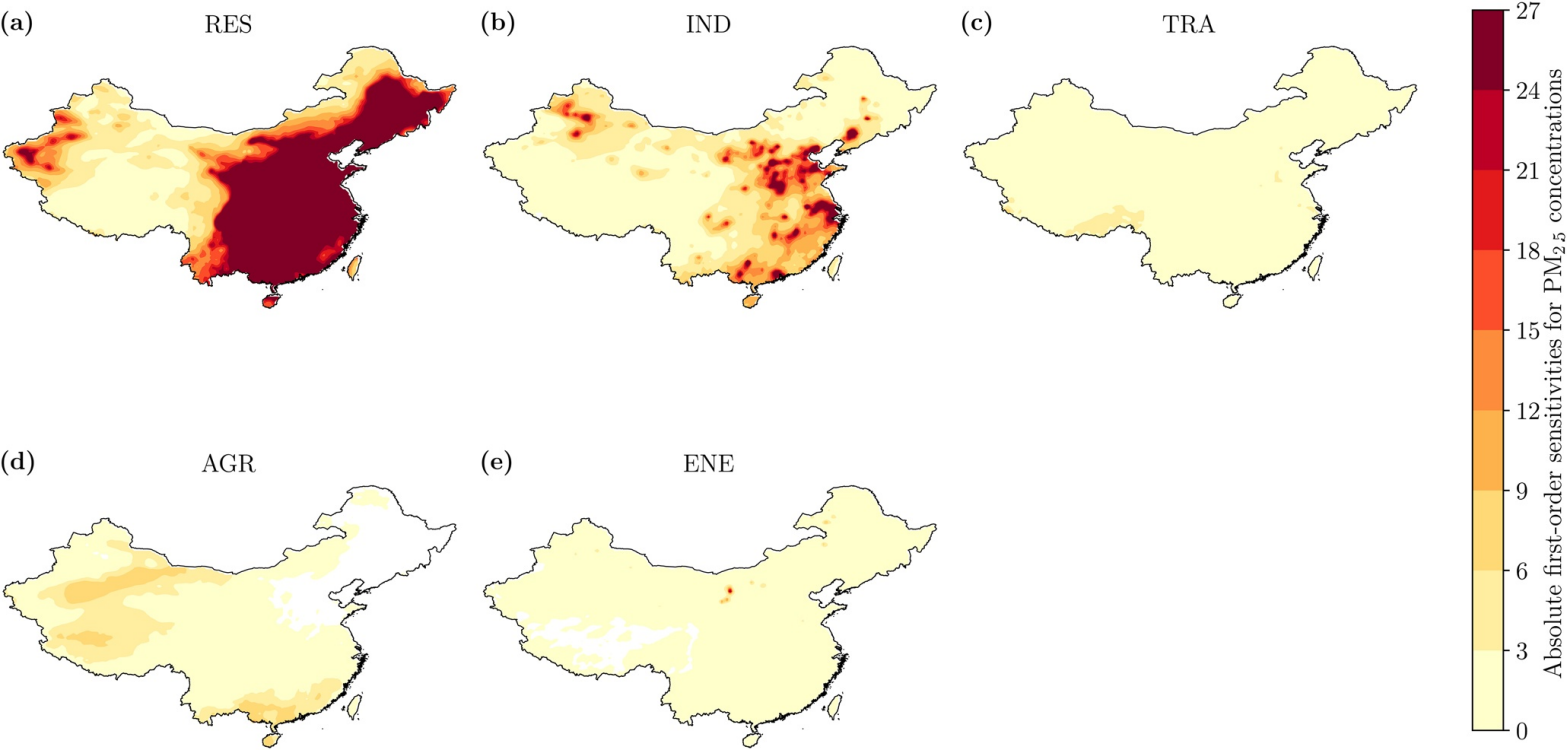
Absolute first‐order sensitivities of monthly‐mean (January 2015) ambient fine particulate matter (PM_2.5_) concentrations across China (first‐order sensitivities multiplied by the baseline PM_2.5_ concentrations). The five key emission sectors are (a) residential (RES), (b) industry (IND), (c) land transport (TRA), (d) agriculture (AGR), and (e) power generation (ENE).

Figure 3 shows the absolute first‐order sensitivities for PM_2.5_ concentrations, which are the first‐order sensitivities multiplied by the baseline PM_2.5_ concentrations. The absolute first‐order sensitivities are largest for residential and industrial emissions. The first‐order sensitivities, which do not account for the magnitude of PM_2.5_ concentrations, are higher for land transport and agricultural emissions in South West China (Figure S5) where PM_2.5_ concentrations are relatively low. All anthropogenic emission sources are lower in South West China compared to other regions, though there are large emissions from the land transport and agricultural sectors in the neighboring regions (Figures S2 and S3) explaining the higher sensitivities.

### 3.3 Impact of Changes in Individual Emission Sectors on Ambient PM_2.5_ Exposure

**Figure 4:**
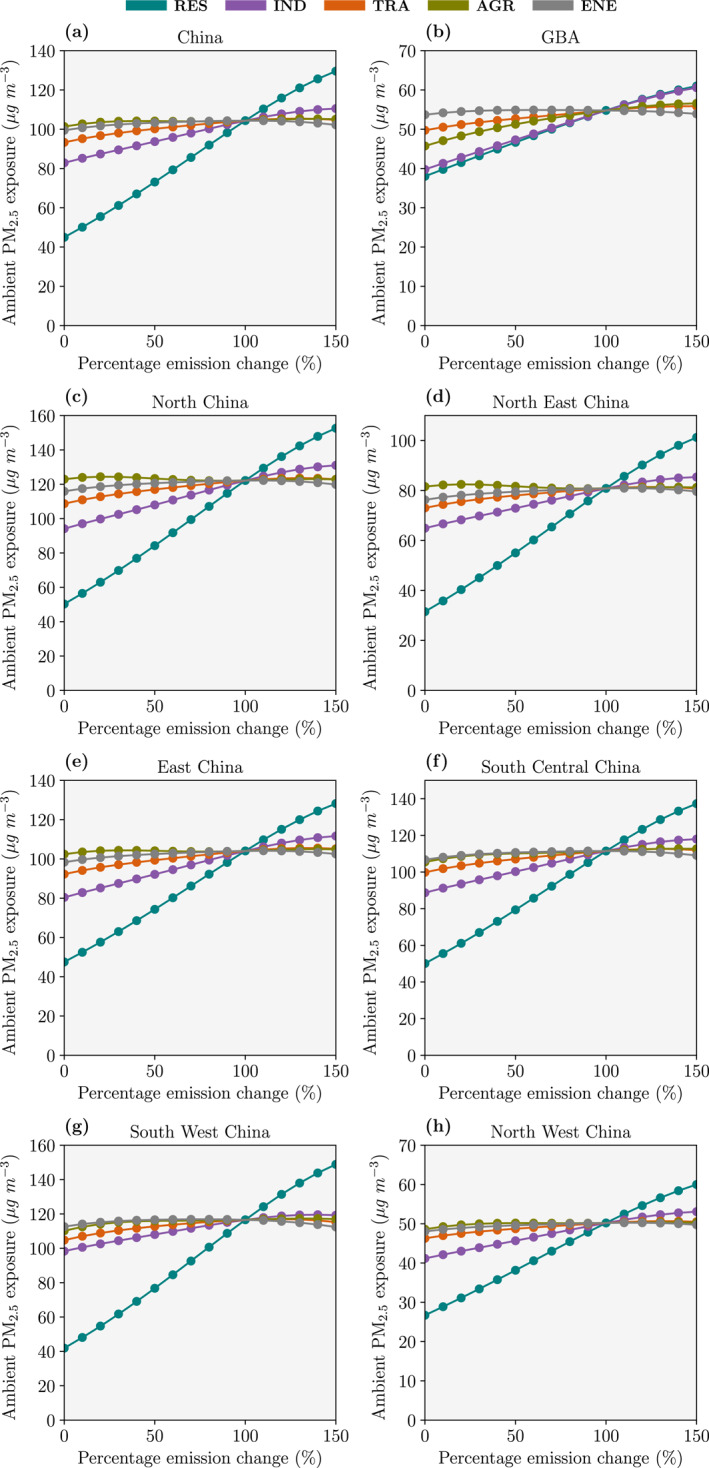
The impact of individual changes in emissions per sector on the monthly‐mean (January 2015) ambient fine particulate matter (PM_2.5_) exposure for (a) China, (b) the Guangdong‐Hong Kong‐Macau Greater Bay Area (GBA), (c) North China, (d), North East China, (e) East China, (f) South Central China, (g) South West China, and (h) North West China for each of the five key emission sectors: residential (RES), industry (IND), land transport (TRA), agriculture (AGR), and power generation (ENE).

The impact of individual changes in emissions, whilst holding other emissions sectors constant at the baseline, on PM_2.5_ exposure are shown in Figure 4. PM_2.5_ exposure can decrease by 31%–64%, down to 26.7–50.3 μg m^−3^. The National Air Quality Target of 35 μg m^−3^ can only be achieved in North West and North East China by reductions in one sector alone. For all regions, reductions in residential emissions produced the largest reduction in PM_2.5_ exposure, followed by industrial emissions. Across China, a 30% reduction in residential emissions reduces PM_2.5_ exposure by 18.8 μg m^−3^ (18%), compared to a 6.3 μg m^−3^ (6%) reduction for a 30% reduction in industrial emissions. Individual changes in land transport, agricultural, and power generation emissions produce smaller impacts on PM_2.5_ exposure. In all regions except the GBA, reductions in land transport emissions produce a larger reduction in PM_2.5_ exposure compared to reductions in agricultural emissions. In the GBA, reductions in agricultural emissions produce a stronger reduction in PM_2.5_ exposure than reductions in land transport emissions. The response in PM_2.5_ exposure from emission changes is approximately linear for all emission sectors.

At the annual timescale, industrial (30%) and residential (26%) emissions dominate the contribution to PM_2.5_ concentrations across China, while there are smaller contributions from agricultural (16%), power generation (14%), and land transport (7%) emissions (Reddington et al., 2019). In January only, the contribution from residential emissions increases to 57% of PM_2.5_ concentrations across China, the contributions from industrial (21%) and land transport (11%) emissions remain similar, and the contributions from agricultural and power generation emissions decrease to 3% and 5%, respectively. Even in the highly urbanized GBA, the contribution from land transport emissions to January PM_2.5_ concentrations is 9% and from agricultural emissions is 17%. These findings suggest that land transport and power generation emissions are not dominant sources of PM_2.5_ concentrations in China. The relatively small contributions of land transport and power generation emissions to regional PM_2.5_ concentrations across China has been found in other modeling studies (GBD MAPS Working Group, 2016; Gu et al., 2018; Hu et al., 2017; Karagulian et al., 2017; Reddington et al., 2019; Shi et al., 2017; Silva et al., 2016) and was further confirmed during the Coronavirus Disease 2019 (COVID‐19) lockdown, when the substantial decreases in land transport and power generation activity only reduced PM_2.5_ concentrations by 10% across China (Silver et al., 2020).

### 3.4 Impact of Changes in Multiple Emission Sectors on Ambient PM_2.5_ Exposure

**Figure 5:**
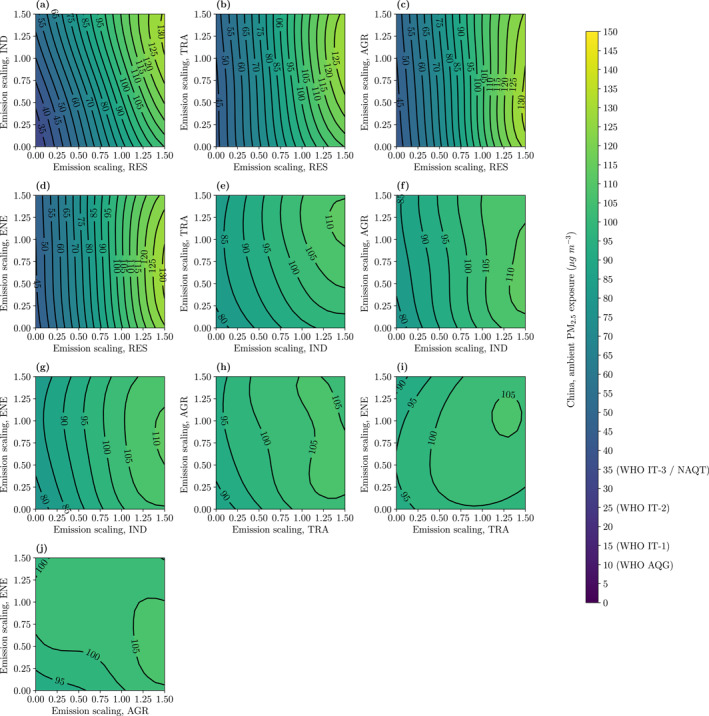
The combined impact of variations in two emission sectors on ambient fine particulate matter (PM_2.5_) exposure for China from (a) residential (RES) and industry (IND), (b) RES and land transport (TRA), (c) RES and agriculture (AGR), (d) RES and power generation (ENE), (e) IND and TRA, (f) IND and AGR, (g) IND and ENE, (h) TRA and AGR, (i) TRA and ENE, and (j) AGR and ENE emissions. Air quality targets shown for the World Health Organization’s (WHO) Air Quality Guideline (AQG), Interim Target 1 (IT‐1), Interim Target 2 (IT‐2), Interim Target 3 (IT‐3), and China’s National Air Quality Target (NAQT).

The combined impacts of variations in two emission sectors on PM_2.5_ exposure for China are shown in Figure 5, and the impacts for specific regions are shown in Figures S6−S12. The greatest reductions in PM_2.5_ exposure are achieved by reducing residential and industrial emissions, with PM_2.5_ exposure going below the National Air Quality Target of 35 μg m^−3^. The combined impacts of reducing residential and industrial emissions on reducing PM_2.5_ exposures are dominated by the reductions in residential emissions for most regions across China (Figure 5), except for the GBA (Figure S6) where there are approximately equal contributions from residential and industrial emissions. Without reducing residential and industrial emissions, reductions in PM_2.5_ exposure across China are limited, with exposure remaining above 90 μg m^−3^ (Figure 5). The exception was for the GBA, where sizable reductions in PM_2.5_ exposure are also obtained from reducing land transport and agricultural emissions together.

Reductions in anthropogenic emissions from the five sectors reduce PM_2.5_ exposure by 68%–81%, down to 15.3–25.9 μg m^−3^. Even when anthropogenic emissions in China from the residential, industrial, land transport, agricultural, and power generation sectors are completely removed, PM_2.5_ exposure remains above the World Health Organization annual guideline of 10 μg m^−3^. In these scenarios, emissions remain from the shipping and aviation sectors, from outside of China, outdoor biomass burning (agricultural and forest fires), and from natural sources such as dust and biogenics. A multi‐model estimate suggests that outdoor biomass burning contributes 4% of annual‐mean PM_2.5_ exposure across China (Reddington et al., 2019). This demonstrates the challenges of reducing winter PM_2.5_ exposure, even under strong national emission reductions. Future work is required to explore the contribution of these different sources.

Our results highlight the importance of reducing residential and industrial emissions in order to reduce winter PM_2.5_ exposure across China, confirming the suggestions of previous studies using different approaches (GBD MAPS Working Group, 2016; Lelieveld et al., 2015; Reddington et al., 2019). The 2018–2020 3‐year plan introduced specific policies for the residential sector in North China in winter to achieve 70% clean heating by 2021 under the Clean Heating Plan (Ministry of Environmental Protection of China, 2017; National Development and Reform Commission of China, 2017). This policy aims to reduce solid fuel use for heating and has the potential to improve air quality and public health in North China (Liu et al., 2019; Meng et al., 2019, 2020; Qin et al., 2017; Zhao et al., 2018). To date there are no specific policies for tackling residential cooking and heating from solid fuels in South China, highlighting a key policy gap that could provide major air quality improvements.

The emulators were trained on simulator data from January 2015. We focused on this period due to the high PM_2.5_ concentrations experienced in winter across China. As the emulators were trained on data from one particular year, they do not account for interannual variability in meteorology, which can impact PM_2.5_ concentrations (Hou et al., 2019; Zhai et al., 2019; Q. Zhang et al., 2019). In future work, we will extend these emulators for long‐term air pollution exposure and the associated chronic disease burden. Here, our focus is on the sensitivity of air pollution to changes in emissions. Different emulators could be developed that explore the sensitivity of air pollution to meteorological variables.

## 4 Conclusion

Air pollution exposure is a leading public health problem in China. Chemical transport models are often used to quantify the impacts of emission changes on air quality. However, the number of sensitivity analyses that can be explored is limited by their large computational demands. Machine learning models can emulate chemical transport models to provide computationally efficient predictions of air quality based on statistical associations with emission changes. Our aim was to develop novel emulators to predict winter ambient PM_2.5_ concentrations from emission changes in five key anthropogenic sectors (residential, industry, land transport, agriculture, and power generation) in China. We used these emulators to explore how PM_2.5_ exposure in January 2015 varied as emissions from the different sectors were varied.

The emulators were based on Gaussian process regressors with Matern kernels. The emulators were optimized to apply Yeo‐Johnson parametric transformations on the inputs, and zero‐mean and unit variance to the transformed outputs. The emulators predicted 99.9% of the variance in PM_2.5_ concentrations for a given input configuration of emission changes. Emulators were developed for each grid cell across China (15,278 grid cells in total) to capture the spatial distribution of PM_2.5_ concentrations.

Global sensitivity analyses were performed on the emulators using a Saltelli sampler and a Sobol analyzer. First‐order sensitivity indices were calculated to measure the contribution that each emission sector makes to the variance within PM_2.5_ concentrations. PM_2.5_ concentrations are primarily sensitive to residential (51%–94% of first‐order sensitivity index), industrial (7%–31%), and agricultural emissions (0%–24%). Sensitivities of PM_2.5_ concentrations to land transport emissions are mostly under 5%, except in South West China where they are 13%. Sensitivities of PM_2.5_ concentrations to power generation emissions are all under 3%.

The emulators were used to predict PM_2.5_ concentrations for every emission configuration of the five anthropogenic emission sectors within a 0%–150% matrix (100% representing January 2015 emissions) at 10% increments. The largest reduction in PM_2.5_ exposure is by 68%–81%, down to 15.3–25.9 μg m^−3^ across China. Even under these emission reduction scenarios, PM_2.5_ exposure exceeds the World Health Organization annual guideline of 10 μg m^−3^. The greatest reductions in PM_2.5_ exposure are all driven by reducing residential and industrial emissions. The annual National Air Quality Target of 35 μg m^−3^ is unlikely to be achieved during winter without stringent emission reductions from the residential and industrial sectors. China is implementing ambitious strategies to reduce emissions in the energy generation, industrial, and land transportation sectors, but until recently there has been less focus on the residential sector (Liu et al., 2016, 2019; Meng et al., 2019, 2020; Zhao et al., 2018). Our work provides further evidence that new policies targeting emission reductions in the residential sector are required if PM_2.5_ exposure across China is to be substantially reduced.

Future work is required to develop these emulators to predict the chronic public health impacts from long‐term air pollution exposure from multiple air pollutants in order to determine the optimum emission reduction strategy to provide the largest improvement in public health.

## Conflict of Interest

The authors declare no conflict of interest relevant to this study.

## Supporting information

Supporting Information S1

## Data Availability

Code to setup and run WRFChem (using WRFotron version 2.0) is available through Conibear and Knote (2020). Emulator code and data is available through Conibear (2020). The trained emulators per grid cell in China that support the findings of this study are available at doi.org/10.5518/953.
