# Supplementary material for: Statistical Emulation of Winter Ambient Fine Particulate Matter Concentrations From Emission Changes in China
Source: Geohealth. 2021 May 1;5(5):e2021GH000391. doi: 10.1029/2021GH000391 (PMC8095364; doi:10.1029/2021GH000391)
Supplement: Supplementary file 1 — Supporting Information S1 [file GH2-5-e2021GH000391-s001.pdf]

AGU PUBLICATIONS

GeoHealth

Supporting information for

Statistical emulation of winter ambient fine particulate matter concentrations from emission changes in China

Luke Conibear[*,1], Carly L. Reddington[1], Ben J. Silver[1], Ying Chen[2], Christoph Knote[3], Stephen R. Arnold[1], and Dominick V. Spracklen[1]

[1] Institute for Climate and Atmospheric Science, School of Earth and Environment, University of Leeds, Leeds, UK

[2] College of Engineering, Mathematics and Physical Sciences, University of Exeter, UK

[3] Faculty of Medicine, University of Augsburg, Germany

* Corresponding author: Luke Conibear (L.A.Conibear@leeds.ac.uk)

Contents of this file

Supplementary Table 1: Simulator setup used in the Weather Research and Forecasting model online–coupled with Chemistry (WRFChem) simulations.

Supplementary Table 2: Scaling factors for each anthropogenic emission sector of the training runs. Applied to all species within the sector. Sectors are residential (RES), industry (IND), land transport (TRA), agriculture (AGR), and power generation (ENE).

Supplementary Table 3: Scaling factors for each anthropogenic emission sector of the testing runs. Applied to all species within the sector. Sectors are residential (RES), industry (IND), land transport (TRA), agriculture (AGR), and power generation (ENE).

Supplementary Figure 1: Anthropogenic emissions of fine particulate matter ($PM_{2.5}$) in January 2015 across China from the (a) residential (RES), (b), industrial (IND), (c) land transport (TRA), and (d) power generation (ENE) sectors.

Supplementary Figure 2: Anthropogenic emissions of ammonia (NH_3) in January 2015 across China from the (a) residential (RES), (b), industrial (IND), (c) land transport (TRA), and (d) agricultural (AGR) sectors.

Supplementary Figure 3: Anthropogenic emissions of nitrogen oxides (NO_X) in January 2015 across China from the (a) residential (RES), (b), industrial (IND), (c) land transport (TRA), and (d) power generation (ENE) sectors.

Supplementary Figure 4: Regional groupings for (a) North China (Beijing, Tianjin, Hebei, Shanxi, and Inner Mongolia), North East China (Liaoning, Jilin, and Heilongjiang), East China (Shanghai, Jiangsu, Zhejiang, Anhui, Fujian, Jiangxi, and Shandong), South Central China (Henan, Hubei, Hunan, Guangdong, Guangxi, Hainan, Hong Kong, and Macau) including the Guangdong−Hong Kong−Macau Greater Bay Area (GBA), South West China (Chongqing, Sichuan, Guizhou, Yunnan, and Tibet), North West China (Shaanxi, Gansu, Qinghai, Ningxia, and Xinjiang), and (b) the GBA individually.

Supplementary Figure 5: First−order sensitivities of monthly−mean (January 2015) ambient fine particulate matter ($PM_{2.5}$) concentrations across China to each of the five key emission sectors: (a) residential (RES), (b) industry (IND), (c) land transport (TRA), (d) agriculture (AGR), and (e) power generation (ENE).

Supplementary Figure 6: The combined impact of variations in two emission sectors on the ambient fine particulate matter ($PM_{2.5}$) exposure for Guangdong−Hong Kong−Macau Greater Bay Area (GBA) from (a) residential (RES) and industry (IND), (b) RES and land transport (TRA), (c) RES and agriculture (AGR), (d) RES and power generation (ENE), (e) IND and TRA, (f) IND and AGR, (g) IND and ENE, (h) TRA and AGR, (i) TRA and ENE, and (j) AGR and ENE emissions. Air quality targets shown for the World Health Organization's

Air Quality Guideline (AQG), Interim Target 1 (IT−1), Interim Target 2 (IT−2), Interim Target 3 (IT−3), and China's National Air Quality Target (NAQT).

Supplementary Figure 7: The combined impact of variations in two emission sectors on the ambient fine particulate matter ($PM_{2.5}$) exposure for North China from (a) residential (RES) and industry (IND), (b) RES and land transport (TRA), (c) RES and agriculture (AGR), (d) RES and power generation (ENE), (e) IND and TRA, (f) IND and AGR, (g) IND and ENE, (h) TRA and AGR, (i) TRA and ENE, and (j) AGR and ENE emissions. Air quality targets shown for the World Health Organization's Air Quality Guideline (AQG), Interim Target 1 (IT−1), Interim Target 2 (IT−2), Interim Target 3 (IT−3), and China's National Air Quality Target (NAQT).

Supplementary Figure 8: The combined impact of variations in two emission sectors on the ambient fine particulate matter ($PM_{2.5}$) exposure for North East China from (a) residential (RES) and industry (IND), (b) RES and land transport (TRA), (c) RES and agriculture (AGR), (d) RES and power generation (ENE), (e) IND and TRA, (f) IND and AGR, (g) IND and ENE, (h) TRA and AGR, (i) TRA and ENE, and (j) AGR and ENE emissions. Air quality targets shown for the World Health Organization's Air Quality Guideline (AQG), Interim Target 1 (IT−1), Interim Target 2 (IT−2), Interim Target 3 (IT−3), and China's National Air Quality Target (NAQT).

Supplementary Figure 9: The combined impact of variations in two emission sectors on the ambient fine particulate matter ($PM_{2.5}$) exposure for East China from (a) residential (RES) and industry (IND), (b) RES and land transport (TRA), (c) RES and agriculture (AGR), (d) RES and power generation (ENE), (e) IND and TRA, (f) IND and AGR, (g) IND and ENE, (h) TRA and AGR, (i) TRA and ENE, and (j) AGR and ENE emissions. Air quality targets shown for the World Health Organization's Air Quality Guideline (AQG), Interim Target 1 (IT−1), Interim Target 2 (IT−2), Interim Target 3 (IT−3), and China's National Air Quality Target (NAQT).

Supplementary Figure 10: The combined impact of variations in two emission sectors on the ambient fine particulate matter ($PM_{2.5}$) exposure for South Central China from (a) residential (RES) and industry (IND), (b) RES and land transport (TRA), (c) RES and agriculture (AGR), (d) RES and power generation (ENE), (e) IND and TRA, (f) IND and AGR, (g) IND and ENE, (h) TRA and AGR, (i) TRA and ENE, and (j) AGR and ENE emissions. Air quality targets shown for the World Health Organization's Air Quality Guideline (AQG), Interim Target 1 (IT−1), Interim Target 2 (IT−2), Interim Target 3 (IT−3), and China's National Air Quality Target (NAQT).

Supplementary Figure 11: The combined impact of variations in two emission sectors on the ambient fine particulate matter ($PM_{2.5}$) exposure for South West China from (a) residential (RES) and industry (IND), (b) RES and land transport (TRA), (c) RES and agriculture (AGR), (d) RES and power generation (ENE), (e) IND and TRA, (f) IND and AGR, (g) IND and ENE, (h) TRA and AGR, (i) TRA and ENE, and (j) AGR and ENE emissions. Air quality targets shown for the World Health Organization's Air Quality Guideline (AQG), Interim Target 1 (IT−1), Interim Target 2 (IT−2), Interim Target 3 (IT−3), and China's National Air Quality Target (NAQT).

Supplementary Figure 12: The combined impact of variations in two emission sectors on the ambient fine particulate matter ($PM_{2.5}$) exposure for North West China from (a) residential (RES) and industry (IND), (b) RES and land transport (TRA), (c) RES and agriculture (AGR), (d) RES and power generation (ENE), (e) IND and TRA, (f) IND and AGR, (g) IND and ENE, (h) TRA and AGR, (i) TRA and ENE, and (j) AGR and ENE emissions. Air quality targets shown for the World Health Organization's Air Quality Guideline (AQG), Interim Target 1 (IT−1), Interim Target 2 (IT−2), Interim Target 3 (IT−3), and China's National Air Quality Target (NAQT).

Additional Supporting Information (Files uploaded separately)

The trained emulators per grid cell in China that support the findings of this study are available at doi.org/10.5518/953.

Supplementary Table 1: Simulator setup used in the Weather Research and Forecasting model online–coupled with Chemistry (WRFChem) simulations.

**Simulator Setup and Parameterisation**	
**Process**	**Method**
**Timestep**	180 seconds.
**Horizontal**	Parent grid on a resolution of 30 km along a 170 × 170 Lambert conformal conical grid, with a 10 km nest over Guangdong–Hong Kong–Macau Greater Bay Area (GBA).
**Vertical**	33 vertical levels, with 38 meteorological levels.
**Microphysics**	Morrison two–moment scheme (Morrison, Thompson, & Tatarskii, 2009).
**Radiation**	Rapid radiative transfer model for general circulation models (RRTMG), short–wave and long–wave (Iacono et al., 2008).
**Boundary layer physics**	Mellor–Yamada Nakanishi and Niino 2.5 (Nakanishi & Niino, 2006).
**Land surface**	Noah Land Surface Model (Ek et al., 2003).
**Convection**	Grell 3–D ensemble (Grell & Devenyi, 2002).
**Gas–phase chemistry**	Extended Model for Ozone and Related Chemical Tracers (MOZART, Emmons et al., 2010; A. Hodzic & Jimenez, 2011; Knote et al., 2014).
**Aerosol**	Updated Model for Simulating Aerosol Interactions and Chemistry (MOSAIC) with aqueous chemistry, volatility basis set secondary organic aerosol production, and 4 sectional bins (Alma Hodzic & Knote, 2014; Knote, Hodzic, & Jimenez, 2015; Zaveri, Easter, Fast, & Peters, 2008).
**Photolysis**	Updated tropospheric ultraviolet-visible (TUV) photolysis based Tie et al., (2003).
**Dust**	Global Ozone Chemistry Aerosol Radiation and Transport (GOCART) with Air Force Weather Agency (AFWA) modifications (Legrand et al., 2019).
**Initial & boundary chemistry**	MOZART / Goddard Earth Observing System (GEOS) Model (National Center for Atmospheric Research, 2016).
**Initial & boundary meteorology**	European Centre for Medium–Range Weather Forecasts (ECMWF) global reanalysis products (Dee et al., 2011).

Supplementary Table 2: Scaling factors for each anthropogenic emission sector of the training runs. Applied to all species within the sector. Sectors are residential (RES), industry (IND), land transport (TRA), agriculture (AGR), and power generation (ENE).

Run	RES	IND	TRA	AGR	ENE
**1**	0.232290	0.205070	0.299040	0.973740	0.551840
**2**	1.121200	1.109800	0.870100	1.222200	1.252000
**3**	0.798430	1.294600	0.090709	0.754010	0.194200
**4**	0.344450	1.422400	0.751120	1.240900	1.211300
**5**	0.625620	1.358900	1.058300	0.046880	0.641960
**6**	1.317300	1.481600	1.260400	0.172230	1.124200
**7**	0.322090	1.224400	1.358300	0.815870	0.881020
**8**	0.504950	1.032500	0.623790	0.346780	0.517600
**9**	1.081500	0.589920	0.174440	0.903560	0.914420
**10**	1.047700	0.852830	1.021800	0.662300	0.295090
**11**	0.200370	0.938770	0.787540	0.840040	0.380760
**12**	0.568470	1.344100	0.243480	1.023100	1.145800
**13**	0.921400	1.452800	1.308100	1.072500	0.976740
**14**	1.057700	0.665280	0.449540	0.899190	0.215160
**15**	0.748300	0.484550	1.404100	0.514490	1.023300
**16**	0.110840	1.074500	0.008116	1.272900	0.110040
**17**	1.159500	1.286100	0.591250	0.747910	0.662940
**18**	0.672560	0.973790	1.148300	0.490410	0.571260
**19**	0.644860	0.133860	1.209700	0.544930	1.362400
**20**	0.017270	0.878860	0.418380	0.623740	0.712510
**21**	0.377570	1.168500	0.991360	0.587440	1.300400
**22**	0.771580	1.244500	1.253700	1.353400	1.450900
**23**	1.372900	0.039672	0.325750	1.316900	0.760170
**24**	0.877930	0.416510	0.374790	0.383360	0.353590
**25**	0.590670	0.384670	1.112800	1.148300	1.480700
**26**	0.868720	1.125300	0.847410	0.011413	1.349800
**27**	0.407450	0.605530	1.423300	0.323490	1.393300
**28**	1.477600	1.196200	0.834050	1.465600	0.161960
**29**	0.269510	0.762190	0.665220	1.095000	0.994200
**30**	0.121540	0.004582	0.971640	0.476100	0.822970
**31**	0.065047	0.920190	0.223860	0.653380	0.148430
**32**	0.516280	0.075462	0.123790	0.414790	0.738230
**33**	1.412200	0.731970	0.486510	0.077169	0.400060
**34**	1.338100	0.434560	0.569770	1.133200	1.186100
**35**	0.292180	0.264880	1.341500	0.713830	0.622110
**36**	0.421000	0.814350	0.076858	0.143540	0.793510
**37**	1.468700	1.001700	0.954640	1.180100	0.848000
**38**	0.969310	0.113760	0.536980	0.807060	1.411400
**39**	0.813420	0.353800	1.470200	1.435800	1.077500
**40**	0.951620	0.278170	0.331140	1.332800	0.265550
**41**	1.409400	0.659160	1.194600	0.276410	1.281200
**42**	1.176900	0.219130	0.461250	0.950480	0.484260
**43**	0.040073	1.399000	0.640110	0.226400	1.095000
**44**	0.464430	0.803670	0.195950	1.013500	0.304760
**45**	0.690130	0.694200	0.692600	0.092788	0.957350
**46**	1.013800	0.172050	1.083200	1.492200	0.002612
**47**	1.256200	0.326790	0.048641	0.260750	0.433230
**48**	0.166260	0.551850	0.908860	1.390900	0.055920
**49**	1.215500	0.476710	0.747650	0.197950	0.075353
**50**	1.285700	0.535410	1.452400	0.444090	0.465490

Supplementary Table 3: Scaling factors for each anthropogenic emission sector of the testing runs. Applied to all species within the sector. Sectors are residential (RES), industry (IND), land transport (TRA), agriculture (AGR), and power generation (ENE).

Run	RES	IND	TRA	AGR	ENE
**1**	0.800999	0.119784	1.370262	0.752336	0.336031
**2**	0.017744	0.523419	0.600339	0.476919	1.439264
**3**	1.005595	1.3623	1.147695	1.144138	1.135136
**4**	1.489352	0.889862	0.530681	0.001446	0.657881
**5**	0.340401	1.107743	0.265786	1.457797	0.181091

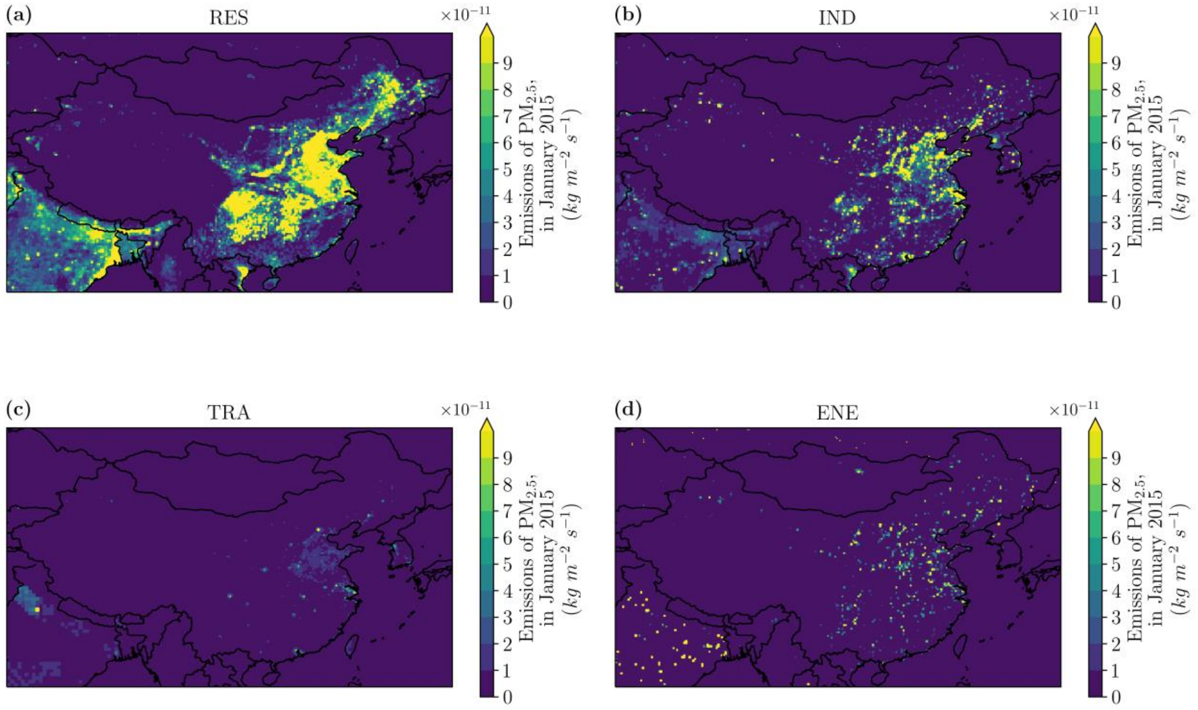

Supplementary Figure 1: Anthropogenic emissions of fine particulate matter ($PM_{2.5}$) in January 2015 across China from the (a) residential (RES), (b), industrial (IND), (c) land transport (TRA), and (d) power generation (ENE) sectors.

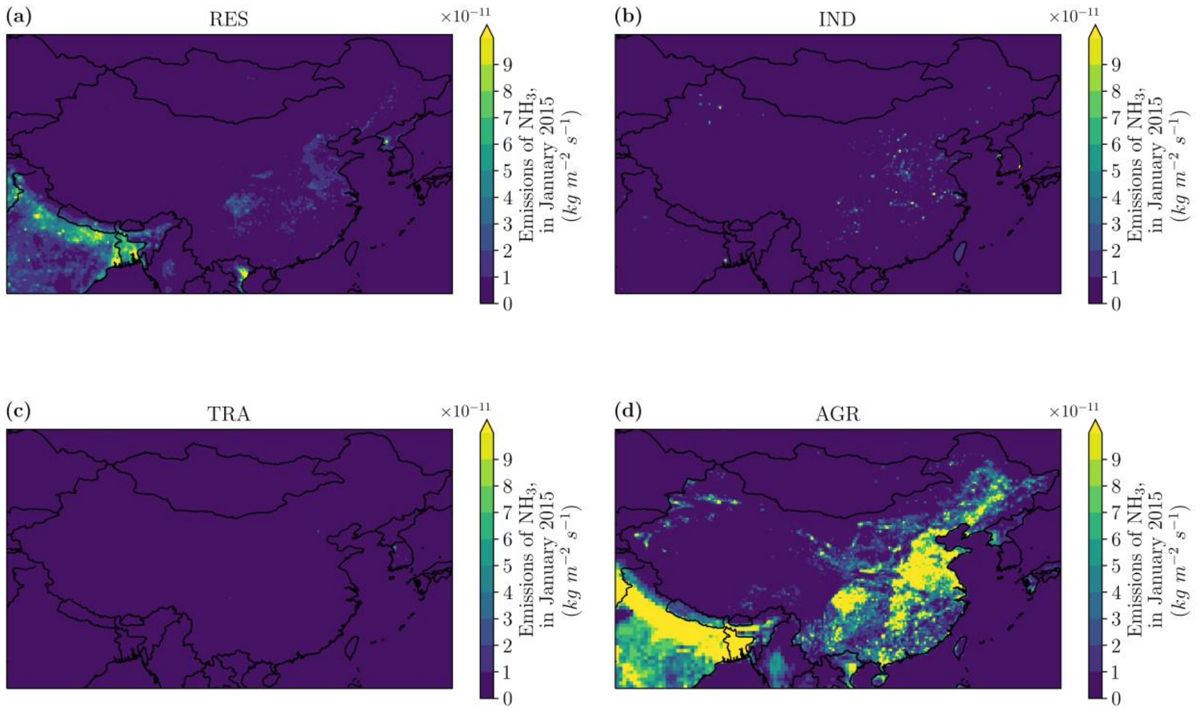

Supplementary Figure 2: Anthropogenic emissions of ammonia (NH_3) in January 2015 across China from the (a) residential (RES), (b), industrial (IND), (c) land transport (TRA), and (d) agricultural (AGR) sectors.

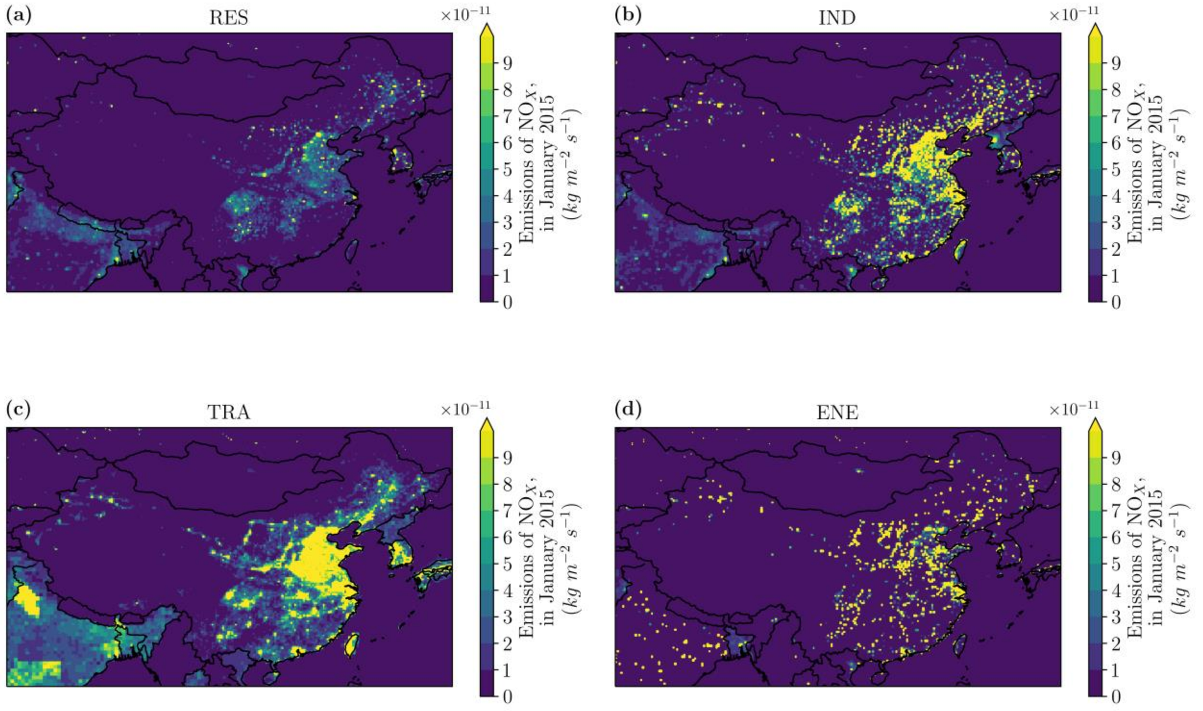

Supplementary Figure 3: Anthropogenic emissions of nitrogen oxides (NO_X) in January 2015 across China from the (a) residential (RES), (b), industrial (IND), (c) land transport (TRA), and (d) power generation (ENE) sectors.

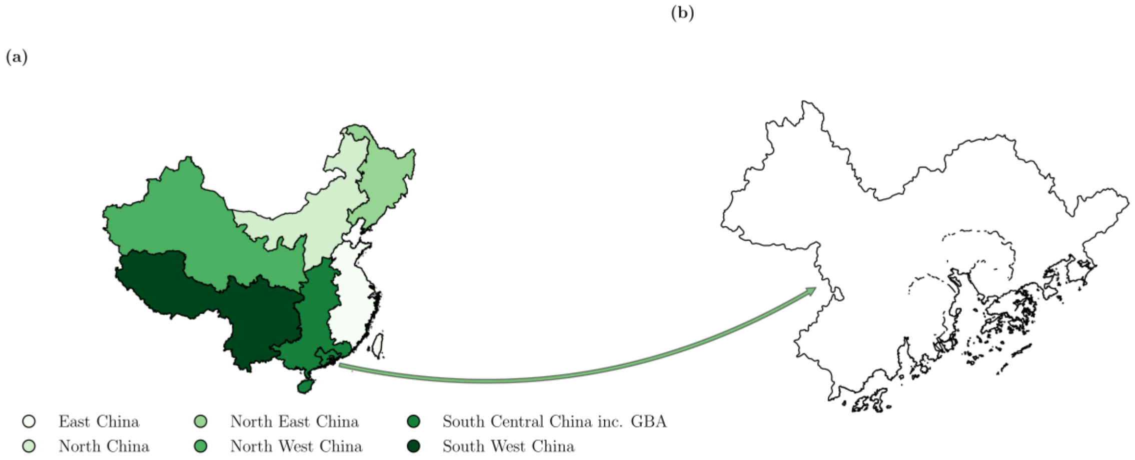

Supplementary Figure 4: Regional groupings for (a) North China (Beijing, Tianjin, Hebei, Shanxi, and Inner Mongolia), North East China (Liaoning, Jilin, and Heilongjiang), East China (Shanghai, Jiangsu, Zhejiang, Anhui, Fujian, Jiangxi, and Shandong), South Central China (Henan, Hubei, Hunan, Guangdong, Guangxi, Hainan, Hong Kong, and Macau) including the Guangdong−Hong Kong−Macau Greater Bay Area (GBA), South West China (Chongqing, Sichuan, Guizhou, Yunnan, and Tibet), North West China (Shaanxi, Gansu, Qinghai, Ningxia, and Xinjiang), and (b) the GBA individually.

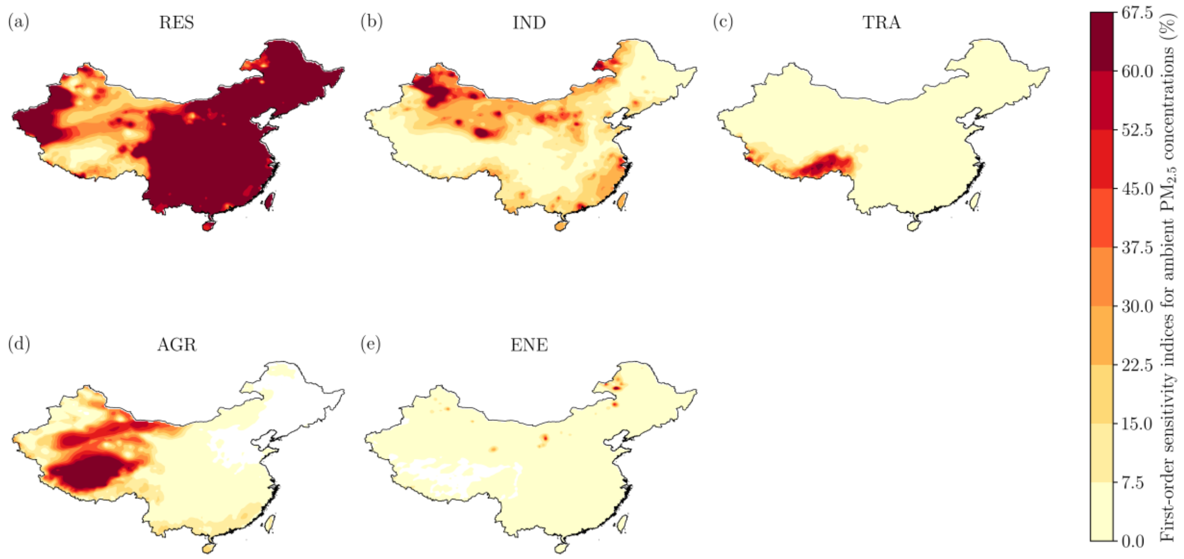

Supplementary Figure 5: First−order sensitivities of monthly−mean (January 2015) ambient fine particulate matter ($PM_{2.5}$) concentrations across China to each of the five key emission sectors: (a) residential (RES), (b) industry (IND), (c) land transport (TRA), (d) agriculture (AGR), and (e) power generation (ENE).

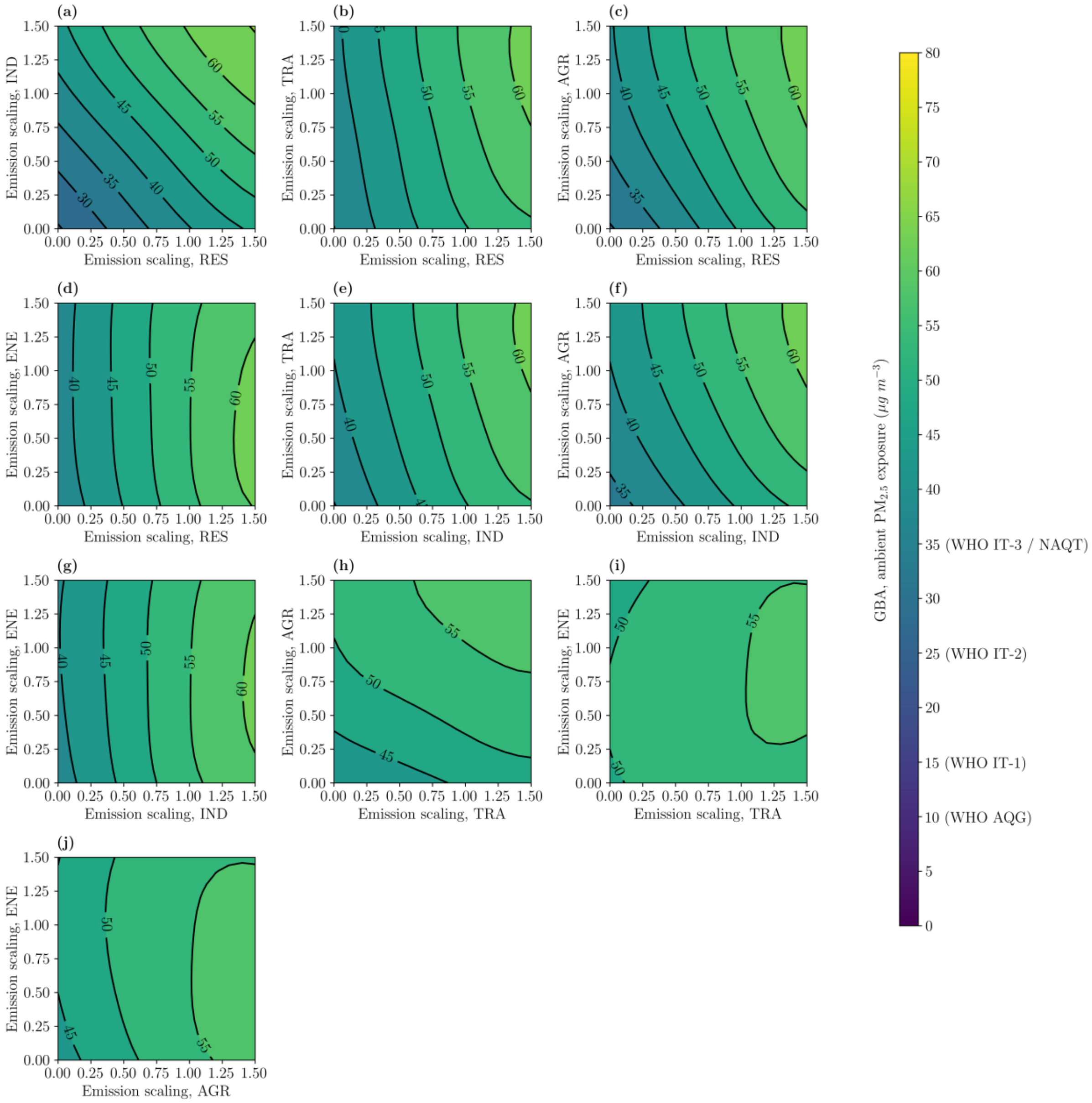

Supplementary Figure 6: The combined impact of variations in two emission sectors on the ambient fine particulate matter ($PM_{2.5}$) exposure for Guangdong−Hong Kong−Macau Greater Bay Area (GBA) from (a) residential (RES) and industry (IND), (b) RES and land transport (TRA), (c) RES and agriculture (AGR), (d) RES and power generation (ENE), (e) IND and TRA, (f) IND and AGR, (g) IND and ENE, (h) TRA and AGR, (i) TRA and ENE, and (j) AGR and ENE emissions. Air quality targets shown for the World Health Organization's Air Quality Guideline (AQG), Interim Target 1 (IT−1), Interim Target 2 (IT−2), Interim Target 3 (IT−3), and China's National Air Quality Target (NAQT).

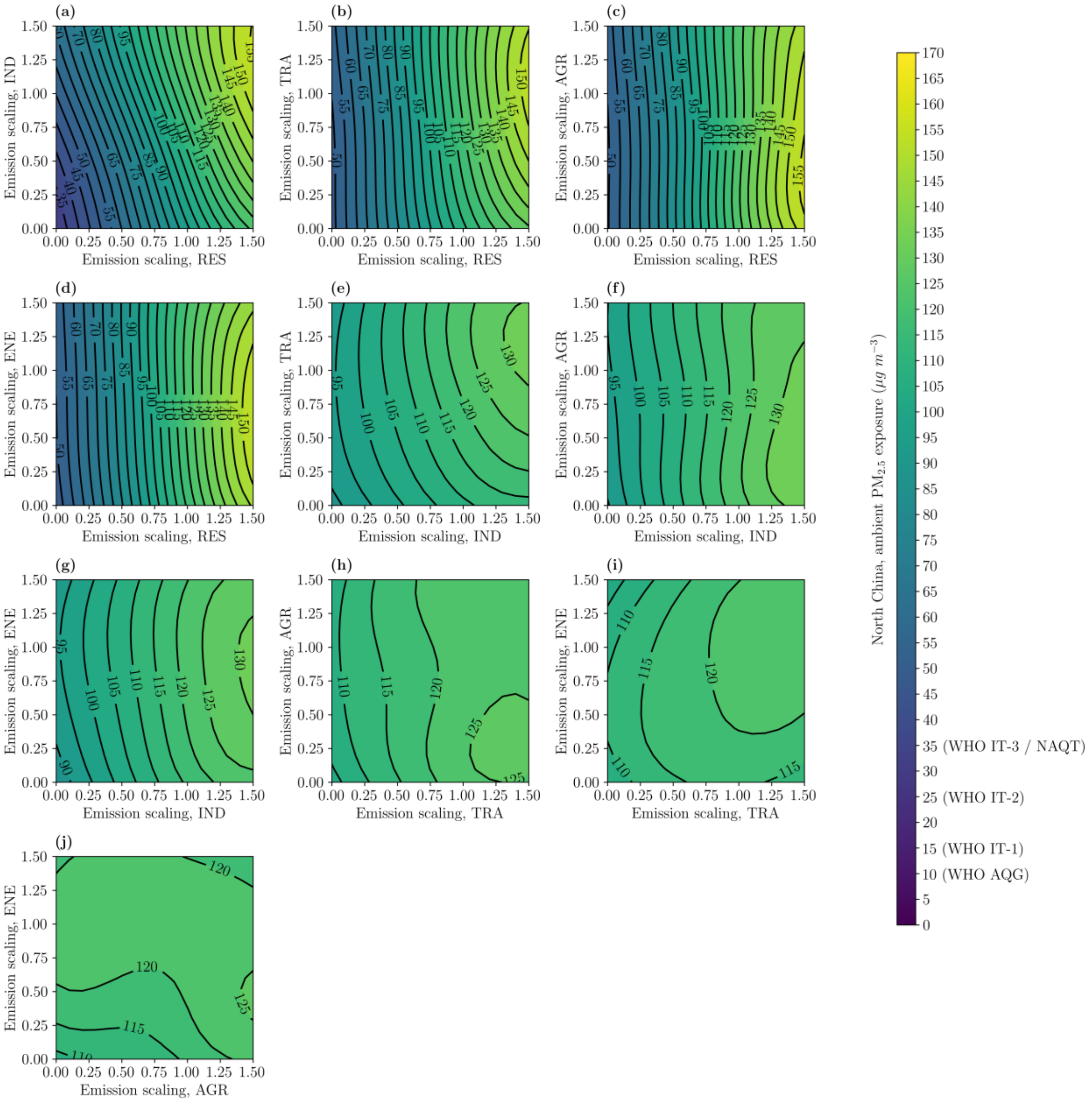

Supplementary Figure 7: The combined impact of variations in two emission sectors on the ambient fine particulate matter ($PM_{2.5}$) exposure for North China from (a) residential (RES) and industry (IND), (b) RES and land transport (TRA), (c) RES and agriculture (AGR), (d) RES and power generation (ENE), (e) IND and TRA, (f) IND and AGR, (g) IND and ENE, (h) TRA and AGR, (i) TRA and ENE, and (j) AGR and ENE emissions. Air quality targets shown for the World Health Organization's Air Quality Guideline (AQG), Interim Target 1 (IT−1), Interim Target 2 (IT−2), Interim Target 3 (IT−3), and China's National Air Quality Target (NAQT).

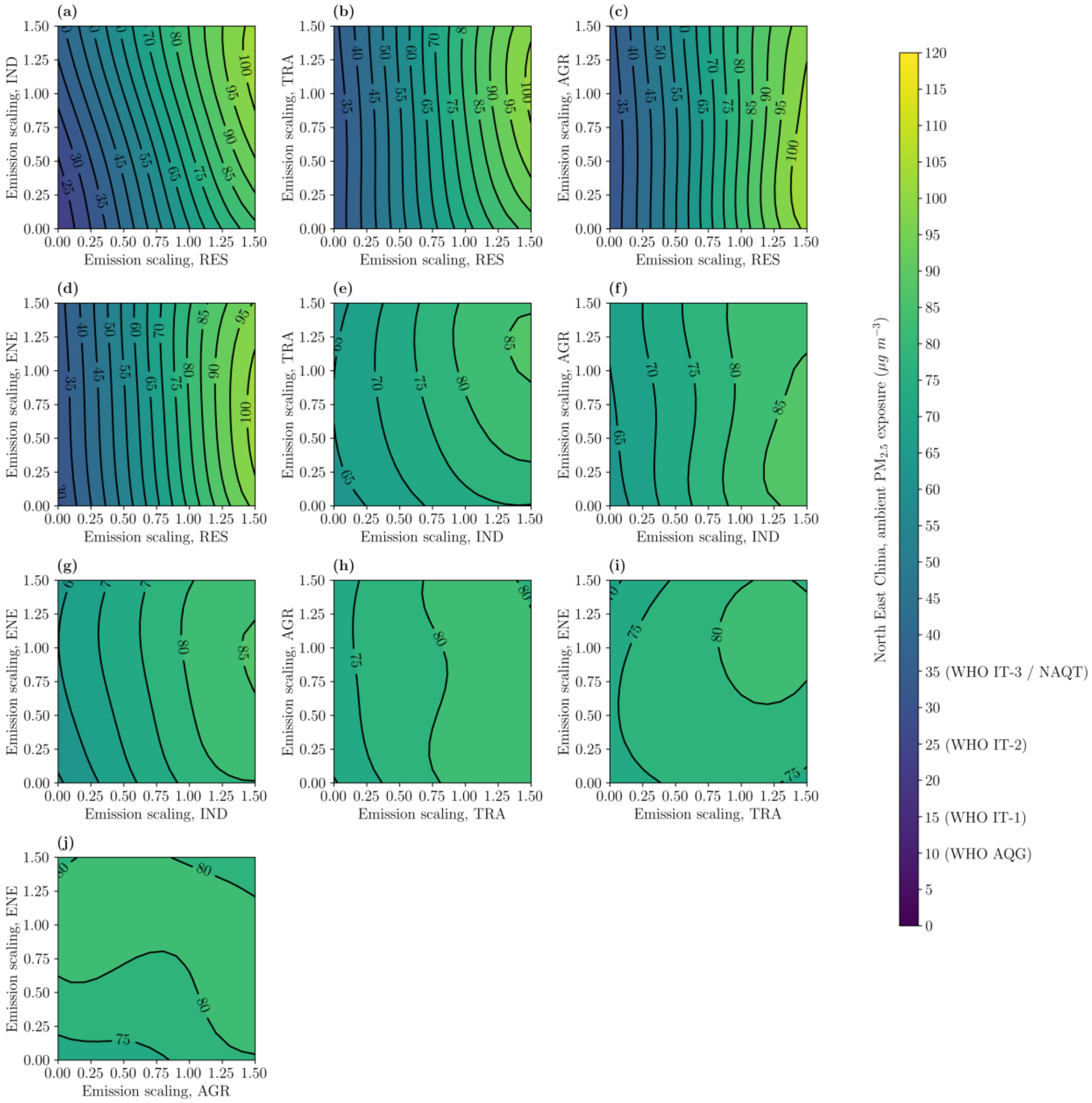

Supplementary Figure 8: The combined impact of variations in two emission sectors on the ambient fine particulate matter ($PM_{2.5}$) exposure for North East China from (a) residential (RES) and industry (IND), (b) RES and land transport (TRA), (c) RES and agriculture (AGR), (d) RES and power generation (ENE), (e) IND and TRA, (f) IND and AGR, (g) IND and ENE, (h) TRA and AGR, (i) TRA and ENE, and (j) AGR and ENE emissions. Air quality targets shown for the World Health Organization's Air Quality Guideline (AQG), Interim Target 1 (IT−1), Interim Target 2 (IT−2), Interim Target 3 (IT−3), and China's National Air Quality Target (NAQT).

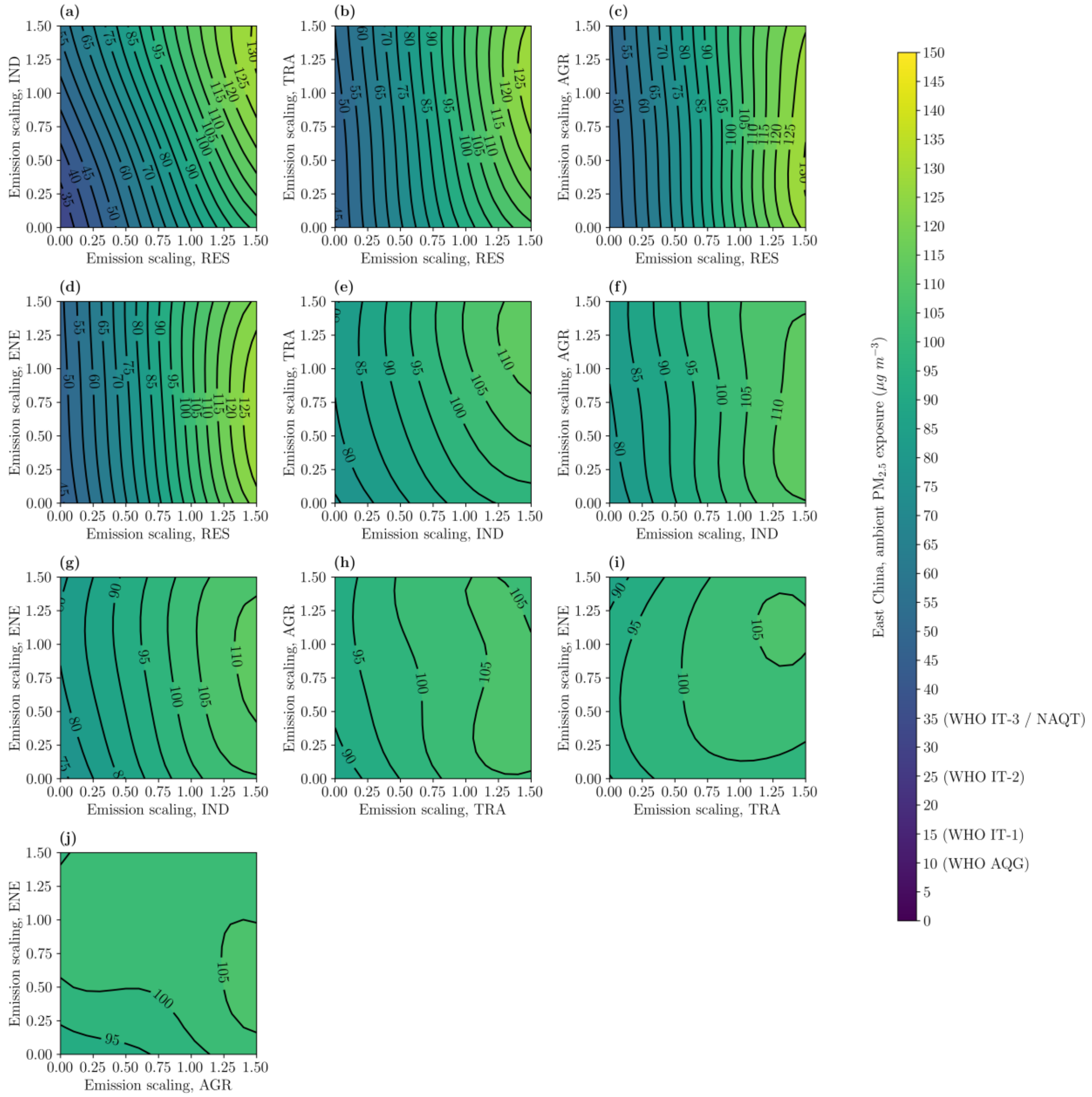

Supplementary Figure 9: The combined impact of variations in two emission sectors on the ambient fine particulate matter ($PM_{2.5}$) exposure for East China from (a) residential (RES) and industry (IND), (b) RES and land transport (TRA), (c) RES and agriculture (AGR), (d) RES and power generation (ENE), (e) IND and TRA, (f) IND and AGR, (g) IND and ENE, (h) TRA and AGR, (i) TRA and ENE, and (j) AGR and ENE emissions. Air quality targets shown for the World Health Organization's Air Quality Guideline (AQG), Interim Target 1 (IT−1), Interim Target 2 (IT−2), Interim Target 3 (IT−3), and China's National Air Quality Target (NAQT).

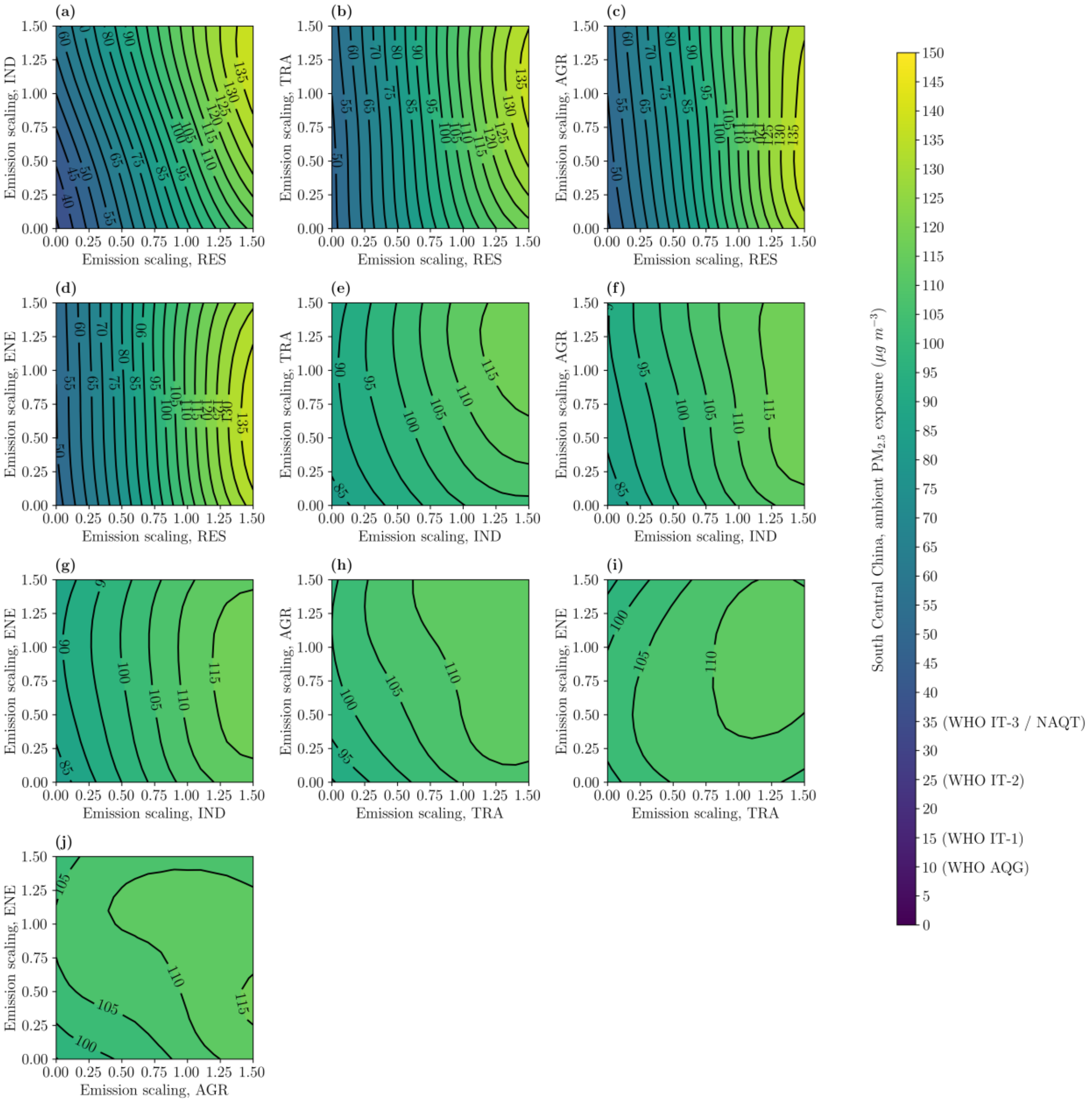

Supplementary Figure 10: The combined impact of variations in two emission sectors on the ambient fine particulate matter ($PM_{2.5}$) exposure for South Central China from (a) residential (RES) and industry (IND), (b) RES and land transport (TRA), (c) RES and agriculture (AGR), (d) RES and power generation (ENE), (e) IND and TRA, (f) IND and AGR, (g) IND and ENE, (h) TRA and AGR, (i) TRA and ENE, and (j) AGR and ENE emissions. Air quality targets shown for the World Health Organization's Air Quality Guideline (AQG), Interim Target 1 (IT−1), Interim Target 2 (IT−2), Interim Target 3 (IT−3), and China's National Air Quality Target (NAQT).

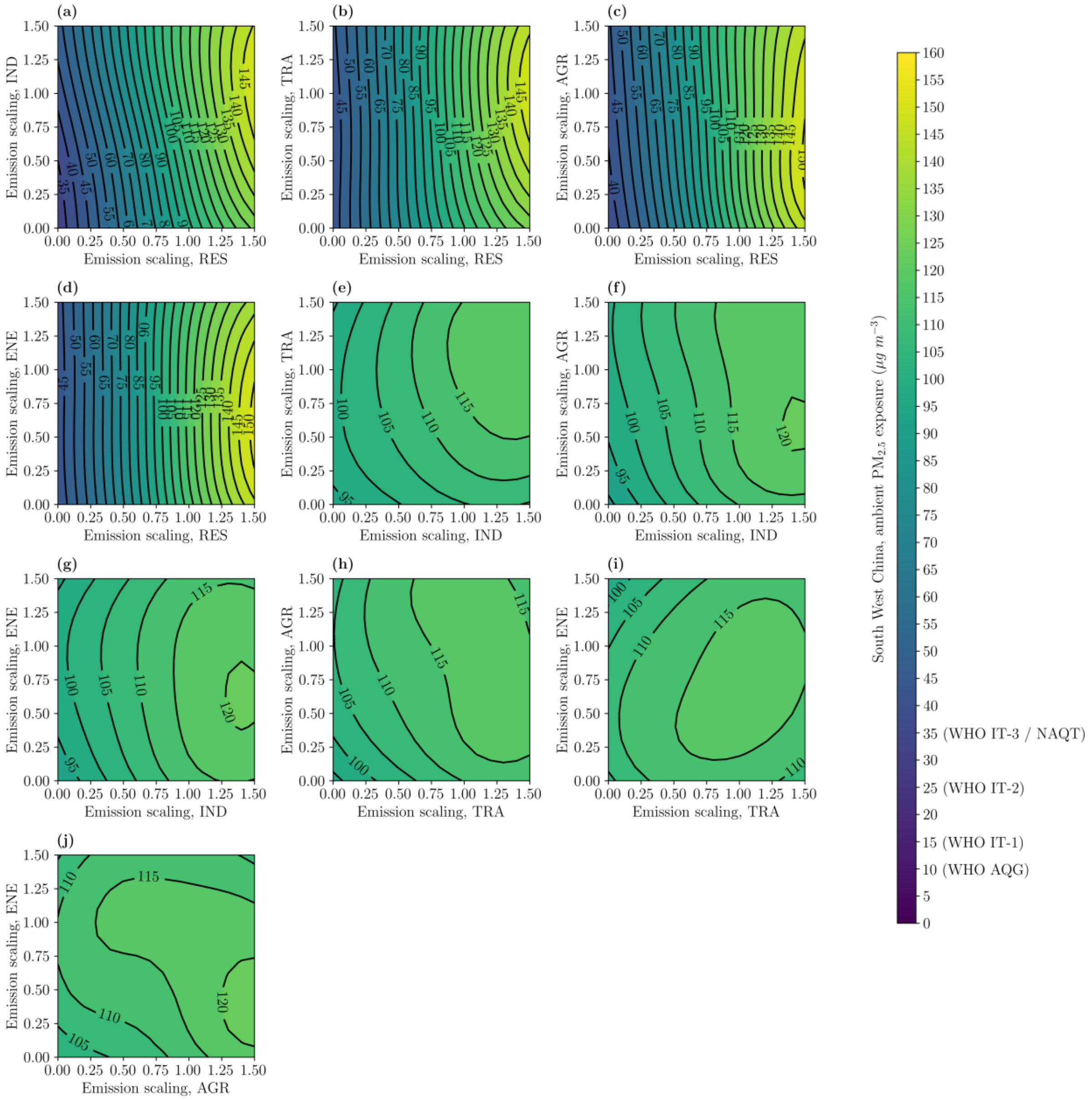

Supplementary Figure 11: The combined impact of variations in two emission sectors on the ambient fine particulate matter ($PM_{2.5}$) exposure for South West China from (a) residential (RES) and industry (IND), (b) RES and land transport (TRA), (c) RES and agriculture (AGR), (d) RES and power generation (ENE), (e) IND and TRA, (f) IND and AGR, (g) IND and ENE, (h) TRA and AGR, (i) TRA and ENE, and (j) AGR and ENE emissions. Air quality targets shown for the World Health Organization's Air Quality Guideline (AQG), Interim Target 1 (IT−1), Interim Target 2 (IT−2), Interim Target 3 (IT−3), and China's National Air Quality Target (NAQT).

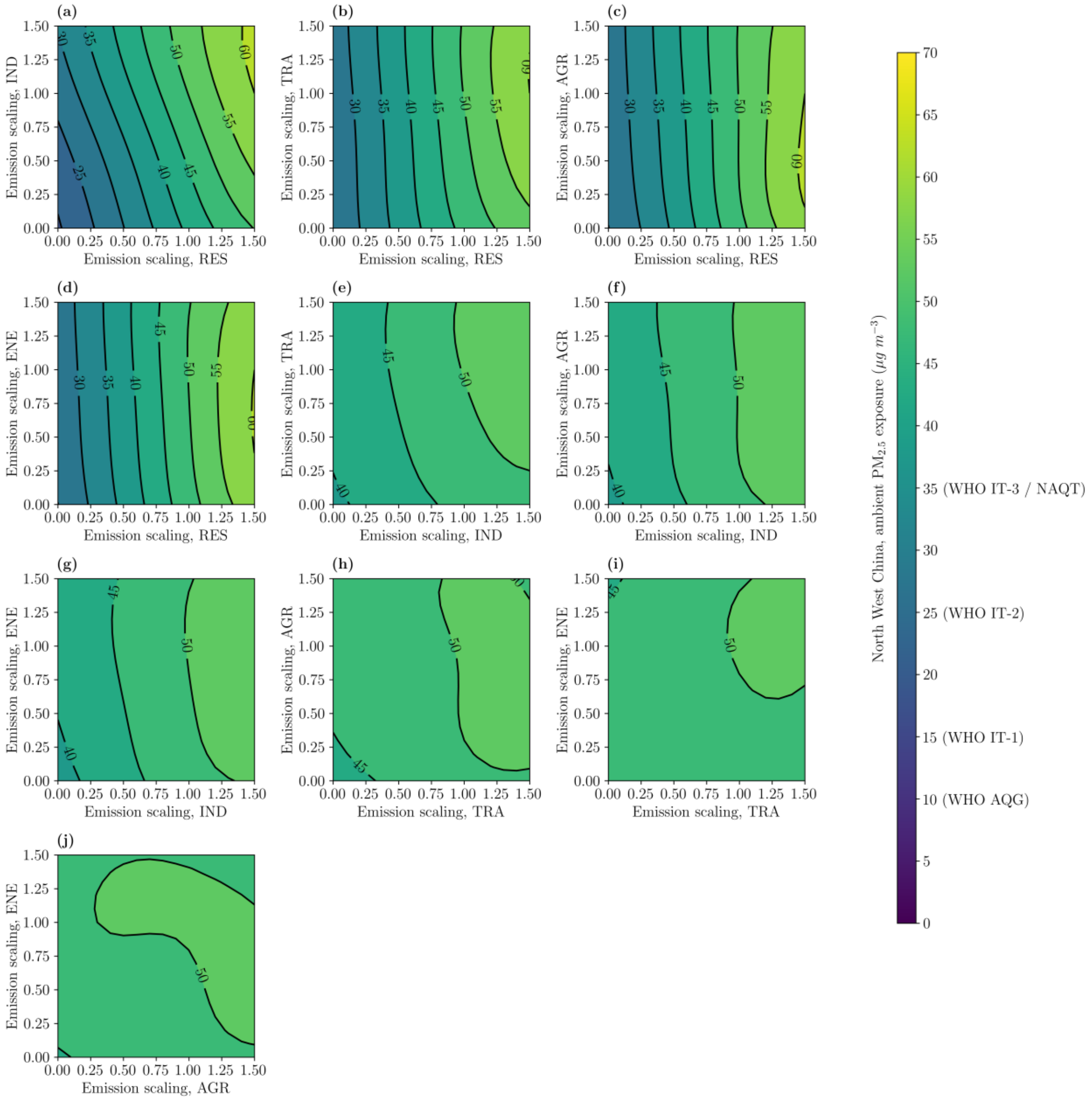

Supplementary Figure 12: The combined impact of variations in two emission sectors on the ambient fine particulate matter ($PM_{2.5}$) exposure for North West China from (a) residential (RES) and industry (IND), (b) RES and land transport (TRA), (c) RES and agriculture (AGR), (d) RES and power generation (ENE), (e) IND and TRA, (f) IND and AGR, (g) IND and ENE, (h) TRA and AGR, (i) TRA and ENE, and (j) AGR and ENE emissions. Air quality targets shown for the World Health Organization's Air Quality Guideline (AQG), Interim Target 1 (IT−1), Interim Target 2 (IT−2), Interim Target 3 (IT−3), and China's National Air Quality Target (NAQT).

References

Dee, D. P., Uppala, S. M., Simmons, A. J., Berrisford, P., Poli, P., Kobayashi, S., et al. (2011). The ERA-Interim reanalysis: Configuration and performance of the data assimilation system. *Quarterly Journal of the Royal Meteorological Society*, *137*(656), 553–597. https://doi.org/10.1002/qj.828

Ek, M. B., Mitchell, K. E., Lin, Y., Rogers, E., Grunmann, P., Koren, V., et al. (2003). Implementation of Noah land surface model advances in the National Centers for Environmental Prediction operational mesoscale Eta model. *Journal of Geophysical Research: Atmospheres*, *108*(D22), 8851–8867. https://doi.org/10.1029/2002JD003296

Emmons, L. K., Walters, S., Hess, P. G., Lamarque, J.-F., Pfister, G. G., Fillmore, D., et al. (2010). Description and evaluation of the Model for Ozone and Related chemical Tracers, version 4 (MOZART-4). *Geoscientific Model Development, 3*, 43–67. https://doi.org//10.5194/gmd-3-43-2010

Grell, G. A., & Devenyi, D. (2002). A generalized approach to parameterizing convection combining ensemble and data assimilation techniques. *Geophysical Research Letters*, *29*(14), 10–13. https://doi.org/10.1029/2002GL015311

Hodzic, A., & Jimenez, J. L. (2011). Modeling anthropogenically controlled secondary organic aerosols in a megacity: a simplified framework for global and climate models. *Geoscientific Model Development*, *4*(4), 901–917. https://doi.org/10.5194/gmd-4-901-2011

Hodzic, Alma, & Knote, C. (2014). WRF-Chem 3.6.1: MOZART gas-phase chemistry with MOSAIC aerosols. *Atmospheric Chemistry Division (ACD), National Center for Atmospheric Research (NCAR)*, 7.

Iacono, M. J., Delamere, J. S., Mlawer, E. J., Shephard, M. W., Clough, S. A., & Collins, W. D. (2008). Radiative forcing by long-lived greenhouse gases: Calculations with the AER radiative transfer models. *Journal of Geophysical Research: Atmospheres*, *113*(13), 2–9. https://doi.org/10.1029/2008JD009944

Knote, C., Hodzic, A., & Jimenez, J. L. (2015). The effect of dry and wet deposition of condensable vapors on secondary organic aerosols concentrations over the continental US. *Atmospheric Chemistry and Physics*, *15*(1), 1–18. https://doi.org/10.5194/acp-15-1-2015

Knote, C., Hodzic, A., Jimenez, J. L., Volkamer, R., Orlando, J. J., Baidar, S., et al. (2014). Simulation of semi-explicit mechanisms of SOA formation from glyoxal in aerosol in a 3-D model. *Atmospheric Chemistry and Physics*, *14*(12), 6213–6239. https://doi.org/10.5194/acp-14-6213-2014

Legrand, S. L., Polashenski, C., Letcher, T. W., Creighton, G. A., Peckham, E., & Cetola, J. D. (2019). The AFWA Dust Emissions Scheme for the GOCART Aerosol Model in WRF-Chem. *Geoscientific Model Development*, *12*, 131–166. https://doi.org/10.5194/gmd-12-131-2019

Morrison, H., Thompson, G., & Tatarskii, V. (2009). Impact of Cloud Microphysics on the Development of Trailing Stratiform Precipitation in a Simulated Squall Line: Comparison of One- and Two-Moment Schemes. *Monthly Weather Review*, *137*(3), 991–1007. https://doi.org/10.1175/2008MWR2556.1

Nakanishi, M., & Niino, H. (2006). An improved Mellor-Yamada Level-3 model: Its numerical stability and application to a regional prediction of advection fog. *Boundary-Layer Meteorology*, *119*(2), 397–407. https://doi.org/10.1007/s10546-005-9030-8

National Center for Atmospheric Research. (2016). ACOM MOZART-4/GEOS-5 global model output. *UCAR*. Retrieved from http://www.acom.ucar.edu/wrf-chem/mozart.shtml

Tie, X., Madronich, S., Walters, S., Zhang, R., Rasch, P., & Collins, W. (2003). Effect of clouds on photolysis and oxidants in the troposphere. *Journal of Geophysical Research*, *108*(D20), 4642, 1–11. https://doi.org/10.1029/2003JD003659

Zaveri, R. A., Easter, R. C., Fast, J. D., & Peters, L. K. (2008). Model for Simulating Aerosol Interactions and Chemistry (MOSAIC). *Journal of Geophysical Research*, *113*(D13204), 1–29. https://doi.org/10.1029/2007JD008782
